# Emergence of human G2P[4] rotaviruses containing animal derived gene segments in the post-vaccine era

**DOI:** 10.1038/srep36841

**Published:** 2016-11-14

**Authors:** Jelle Matthijnssens, Valerie Nuyts, Elisabeth Heylen, Sarah De Coster, Nádia Conceição-Neto, Marc Van Ranst, Jelle Matthijnssens

**Affiliations:** 1 KU Leuven - University of Leuven, Department of Microbiology and Immunology, Laboratory for Clinical and Epidemiological Virology, Rega Institute for Medical Research, Leuven, Belgium; 2 KU Leuven - University of Leuven, Department of Microbiology and Immunology, Laboratory of Viral Metagenomics, Rega Institute for Medical Research, Leuven, Belgium

## Abstract

The introduction of Rotarix into the Belgian immunization program in June 2006 coincided with an increase of the relative prevalence of G2P[4] strains. However, the genetic composition of these persistent G2P[4] strains has not been investigated. Therefore, we have investigated the NSP4 gene of 89 Belgian G2P[4] strains detected between 1999 and 2013, covering both pre- and post-vaccination periods. The NSP4 genes were divided over seven separate clusters of which six were more closely related to animal than to human strains. The NSP4 genes that clustered more closely to animal DS-1-like strains were isolated after 2004–2005 and were found throughout multiple seasons. Complete genome sequencing of 28 strains identified several other gene segments that clustered more closely to animal than to human DS-1-like strains. These findings suggest that frequent interspecies reassortments may have played a role in the spread of G2P[4] rotaviruses in the post-vaccination period in Belgium.

Group A rotaviruses are the most important etiological agent for gastroenteritis in children under 5 years of age^1^, and possess a triple layer protein shell that consists of six structural proteins (VP1-4, VP6 and VP7). Besides structural proteins the rotavirus genome also encodes 6 non-structural proteins (NSP1-6)^2^. The segmented nature of the rotavirus genome allows for gene reassortment which is considered an important evolutionary mechanism for rotaviruses to generate genetic diversity by, for example, introducing rotavirus gene segments from one animal species into the rotavirus population of another species^3^,^4^,^5^. Complete genome analysis of human rotaviruses has revealed the existence of two major genotype constellations: The Wa-like genotype constellation consists of a genotype 1 genetic background (I1-R1-C1-M1-A1-N1-T1-E1-H1) and is found in combination with a P[8] VP4 and many different G-genotypes (G1, G3, G4, G9 and G12)^6^. The DS-1-like genotype constellation is the second major genotype constellation and is characterized by a genotype 2 genotype constellation except for VP4 which is typically a P[4] (G2-P[4]-I2-R2-C2-M2-A2-N2-T2-E2-H2)^6^. Both genotype constellations have distinct evolutionary origins: Most gene segments of Wa-like rotaviruses are thought to share a common ancestor with porcine rotaviruses, while most gene segments of DS-1-like rotaviruses likely have a common origin with bovine(-like) rotaviruses^6^,^7^.

DS-1-like rotaviruses are less prevalent than their Wa-like counterparts and therefore relatively little is known about their long-term evolution^6^. Their common ancestry with bovine rotaviruses facilitates the possibility of interspecies reassortment with bovine rotaviruses as the VP1-VP3,VP6, NSP2 and NSP4 gene segments of human DS-1-like rotaviruses and bovine rotaviruses almost always share the same genotype^7^. In addition, even though bovine-like rotaviruses possessing a P[14] VP4 are infrequently detected, they have been described in humans on several occasions, which further facilitates interspecies reassortment between human and bovine rotaviruses^8^,^9^. However, it is currently unknown to what extent these interspecies reassortments occur as the number of completely sequenced DS-1-like genomes is limited. The analysis of complete DS-1-like genomes isolated in Brazil, Italy and the USA revealed the co-circulation of multiple lineages for several gene segments, some of which are likely of animal origin^10^,^11^,^12^. In addition, the circulation of animal-human reassortant viruses possessing an animal NSP4 gene segment has been increasingly reported^13^,^14^,^15^,^16^. Animal-human reassortant viruses have the potential to change viral fitness and promote viral transmission, especially in an immune naïve population. Previously, this was observed for G9 and G12 Wa-like rotaviruses, possessing VP7 genes that are thought to have originated from porcine rotaviruses and have efficiently spread globally in the human population in less than two decades^17^.

In Belgium, Rotarix was introduced in 2006 and RotaTeq in 2007. Approximately 85% of all administered rotavirus vaccines in Belgium is Rotarix. The introduction of Rotarix coincided with an increased relative prevalence of G2P[4] rotaviruses, which was sustained throughout multiple seasons^18^. Although it was previously shown that the vaccine effectiveness of Rotarix against DS-1-like G2P[4] strains was slightly lower than against Wa-like strains^19^,^20^,^21^, we hypothesize that also changes in the DS-1-like rotaviruses themselves, may have influenced the sudden increase in the relative prevalence of DS-1-like rotaviruses in Belgium. To determine to what extent reassortment had occurred in Belgian DS-1-like rotaviruses we analyzed 89 NSP4 genes and 28 complete genomes of Belgian DS-1-like rotaviruses that were isolated before and after vaccine introduction.

## Results

### Viral dataset

Based on a preliminary phylogenetic analysis of VP7, a selection was made of Belgian G2P[4] rotaviruses collected between 1999 and 2013. In total, 89 strains were selected and the complete NSP4 gene was sequenced. NSP4 sequences were obtained from every rotavirus season between 1999 and 2013 except for the 2002–2003 rotavirus season. Despite repeated attempts no reliable sequences could be obtained for the few available strains from 2002–2003, possibly due to low viral load.

### Interspecies reassortment of NSP4 is common in Belgian G2P[4] rotaviruses

**Figure 1:**
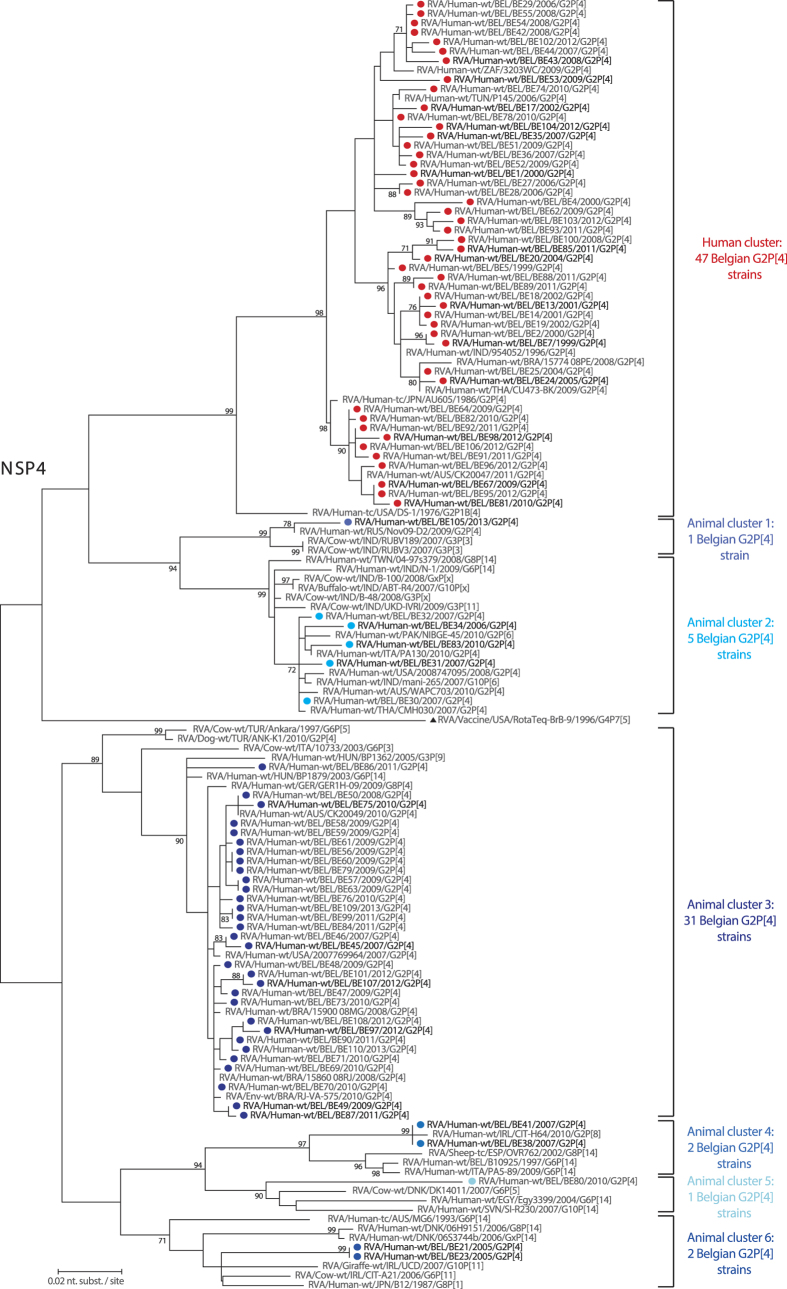
Maximum likelihood tree of the NSP4 gene. Belgian G2P[4] rotaviruses were found in seven distinct clusters (one human cluster (red) and six animal clusters (shades of blue)). Strains analyzed in this study are indicated with a filled circle. The RotaTeq vaccine NSP4 is indicated by the black triangle. Selected strains for complete genome sequencing are highlighted in bold face. Only bootstrap values above 70% are shown.

The 89 Belgian NSP4 sequences were compared with DS-1-like strains deposited in GenBank (Fig. 1) and were found in seven distinct clusters, which were between 10.1% and 15.1% different on the nucleotide level. A little more than half of the G2P[4] strains (n = 47; indicated by red circles in Fig. 1) were found in a cluster together with other typical human DS-1-like strains isolated from various places around the world. The remaining 42 Belgian G2P[4] strains were either clustering more closely to bovine or bovine-like human strains or were clustering relatively distantly from any other known strain (indicated by circles in different shades of blue). The largest of these clusters (animal cluster 3) comprised 31 Belgian G2P[4] strains, which were all detected between 2008 and 2013. They showed on average only 1.9% nucleotide diversity, indicating sustained human-to-human transmission instead of independent interspecies transmission events. These strains were most closely related to environmental and human G2P[4] strains from Brazil, Germany, Australia and the USA. Clustering slightly more distantly to these, there were two animal derived human strains from Hungary, RVA/Human-wt/HUN/BP1879/2003/G6P[14] and RVA/Human-wt/HUN/BP1362/2005/G3P[9], which are thought to be of bovine(-like) and feline origin, respectively. Finally, clustering even more distantly, at approximately 6% nucleotide difference, there were bovine and canine rotaviruses isolated in Turkey and Italy, suggesting that this large cluster of human E2 strains may have originated in animals.

The third largest cluster (animal cluster 2) contained five closely related Belgian G2P[4] strains detected between 2006 and 2010 that were probably the result of human-to-human transmission events and were intermingling with other human G2 and G12 strains isolated from the USA, Thailand, India, Pakistan and Australia. Close to this cluster at 1.0–1.6% nucleotide distance a group of bovine rotaviruses isolated in India was found as well as two bovine-like human P[14] strains, RVA/Human-wt/TWN/04-97s379/2008/G8P[14] and RVA/Human-wt/IND/N-1/2009/G6P[14] (Fig. 1).

RVA/Human-wt/BEL/BE21/2005/G2P[4] and RVA/Human-wt/BEL/BE23/2005/G2P[4] were relatively distantly related to other rotaviruses (animal cluster 6), but most closely to bovine-like human strains isolated from Denmark, Australia and Japan and animal strains from Ireland (between 5.3–9.4% nucleotide difference). RVA/Human-wt/BEL/BE38/2007/G2P[4] and RVA/Human-wt/BEL/BE41/2007/G2P[4] (animal cluster 4) were almost identical to RVA/Human-wt/IRL/CIT-H64/2010/G2P[8], and approximately 4.9–6.5% divergent from a group of bovine-like P[14] strains isolated from humans and a sheep in Italy, Belgium and Spain. Two distinct Belgian G2P[4] strains, RVA/Human-wt/BEL/BE80/2010/G2P[4] and RVA/Human-wt/BEL/BE105/2012/G2P[4] (animal cluster 1 and 5), did not cluster together with any other Belgian G2P[4] strains. RVA/Human-wt/BEL/BE80/2010/G2P[4] was most closely related to a bovine-like human strain isolated from Egypt, but still 7.1% divergent on the nucleotide level, followed by bovine and bovine-like human strains from Denmark and Slovenia showing 7.6% and 9.2% nucleotide difference, respectively. RVA/Human-wt/BEL/BE105/2012/G2P[4] was 1.4% different from RVA/Human-wt/RUS/Nov09-D2/2009/G2P[4], and 2.7% different from two bovine rotaviruses from India. None of the Belgian G2P[4] strains possessed a NSP4 gene segment that clustered closely to the RotaTeq vaccine strain (Fig. 1).

**Figure 2:**
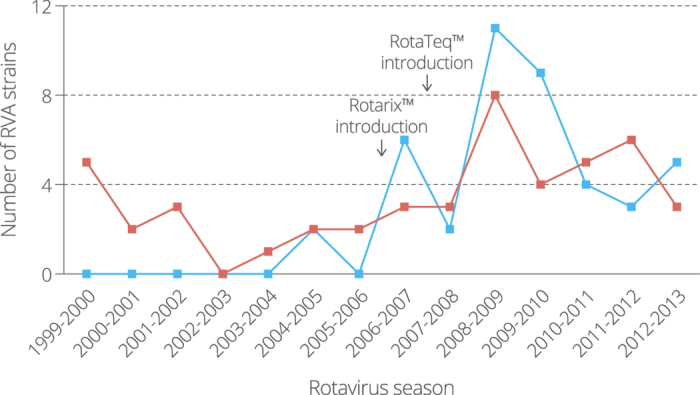
Temporal distribution of 89 Belgian NSP4 genes which are most closely related to typical human NSP4 genes (red) or to bovine and/or bovine-like human NSP4 genes (blue).

Based on the phylogenetic analysis, Belgian NSP4 genes were classified according to their most likely origin: human (n = 47; 52.8%) or animal (n = 42; 47.2%). The temporal distribution of the Belgian bovine and human NSP4 genes showed that before the introduction of Rotarix in Belgium bovine NSP4 genes were only sporadically detected (only 2 out of 17 strains; 11.7%) (Fig. 2). The two bovine-like NSP4 genes detected before vaccine introduction (animal cluster 6) formed a single cluster and probably represented a single interspecies reassortment event. After vaccine introduction bovine-like NSP4 genes constituted 56% of all NSP4 genes (40 out of 72 strains) in our sample set, including both clusters were human-to-human transmission had occurred (animal clusters 3 and 4).

### Complete genome analysis reveals zoonotic reassortments in gene segments other than NSP4

**Figure 3:**
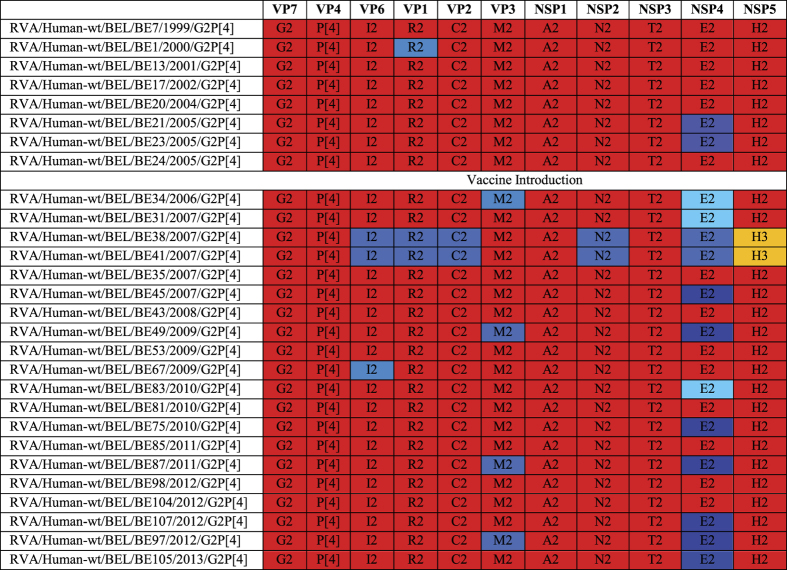
Genotype constellations of 28 Belgian G2P[4] rotaviruses. Human DS-1-like gene segments are indicated in red, whereas DS-1-like gene segments more closely related to bovine strains are indicated in different shades of blue. AU-1-like gene segments are indicated in orange.

Twenty-eight strains were selected for complete genome sequencing based upon the genetic diversity of NSP4, the year of sample isolation and availability of sufficient stool sample (Fig. 3). Analysis with the RotaC online genotyping tool^22^ showed that 26 out of 28 Belgian rotaviruses possessed a typical DS-1-like genotype constellation (G2-P[4]-I2-R2-C2-M2-A2-N2-T2-E2-H2). Two other strains, BE38 and BE41 possessed an H3 NSP5, but contained DS-1-like genotypes for the remaining gene segments (G2-P[4]-I2-R2-C2-M2-A2-N2-T2-E2-H3). Phylogenetic analyses were performed for VP1-VP4, VP6, VP7, NSP1-NSP3 and NSP5 to determine the most likely origin (human or animal) for each DS-1-like gene segment.

**Figure 4:**
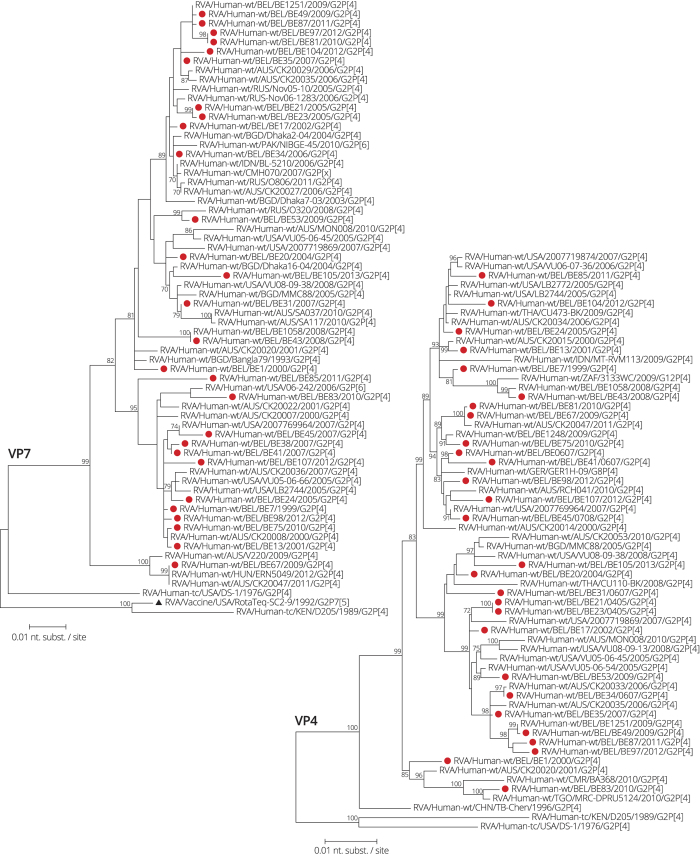
Maximum likelihood tree of VP7 and VP4. Belgian G2P[4] rotaviruses are indicated with a filled red circle. The RotaTeq vaccine strain is indicated by a black triangle. Only bootstrap values above 70% are shown.

**Figure 5:**
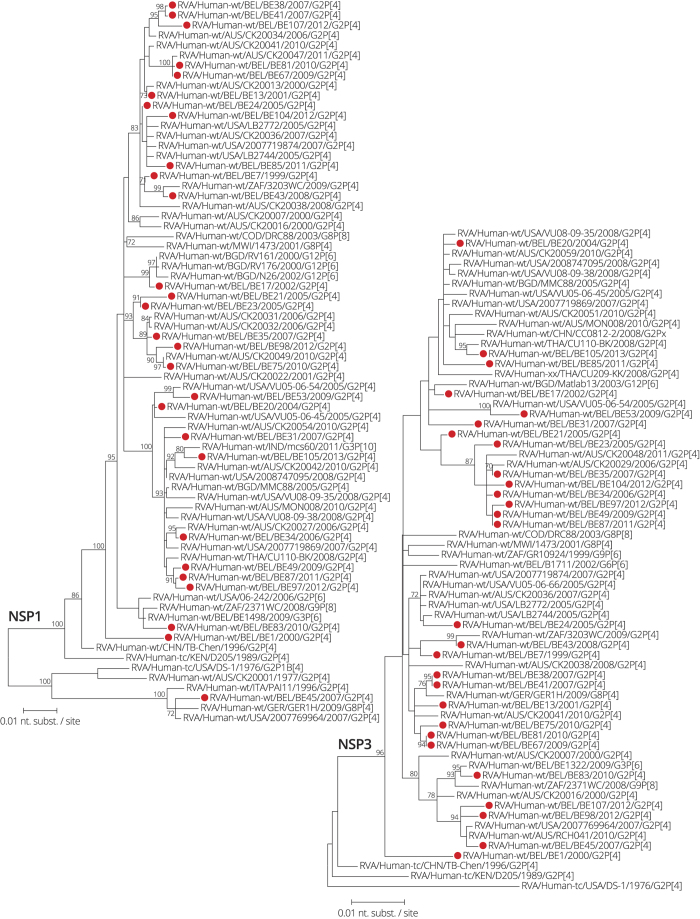
Maximum likelihood tree of NSP1 and NSP3. Belgian G2P[4] rotaviruses are indicated with a filled red circle. Only bootstrap values above 70% are shown.

**Figure 6:**
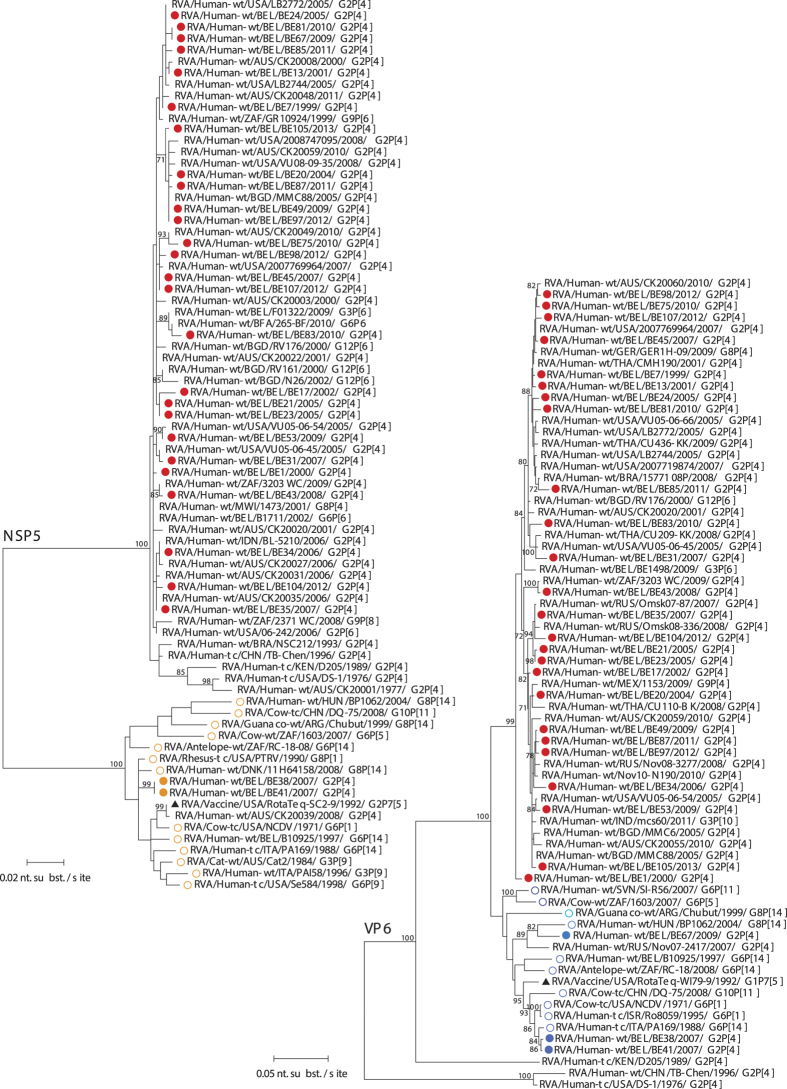
Maximum likelihood tree of NSP5 and VP6. Belgian G2P[4] rotaviruses are indicated with a filled circle and colored red when clustering most closely to human DS-1-like strains and colored in different shades of blue when clustering most closely to bovine or bovine-like human rotaviruses. Orange circles indicate AU1-like genotypes. Bovine or bovine-like human reference strains are indicated with an open circle in different shades of blue. The RotaTeq vaccine strain is indicated by a black triangle. Only bootstrap values above 70% are shown.

The phylogenetic tree of VP7 showed that Belgian strains were found in three clusters together with contemporary human G2P[4] and G2P[6] strains isolated worldwide (Fig. 4). One Belgian strain, RVA/Human- wt/BEL/BE67/2009/G2P[4], was more distantly related to the other Belgian G2P[4] rotaviruses (95.2-96.5% similarity on the nucleotide level). None of the Belgian strains clustered closely to the G2 component of the RotaTeq vaccine (6.2–7.8% difference). The VP4, NSP1 and NSP3 genes of the 28 Belgian strains clustered closely together with other contemporary human DS-1-like rotaviruses although for every gene segment one or two exceptions could be identified (Figs 4 and 5). In particular in the NSP1 tree, strain RVA/Human-wt/BEL/BE45/2007/G2P[4] was found to be 7.1–8.5% divergent from other Belgian DS-1-like NSP1 genes. The Belgian DS-1-like strains belonging to the H2 genotype showed only little diversity (<2.4%) (Fig. 6). The two strains belonging to the H3 genotype, BE38 and BE41, were identical and closely related with RVA/Rhesus-tc/USA/PTRV/1990/G8P[1], which has previously been characterized as an interspecies transmission from a ruminant to a monkey^4^,^23^, and the bovine-like human strain RVA/Human-wt/DNK/11H64158/2008/G8P[14]. Even though NSP5 is not a genetically diverse gene, the RotaTeq NSP5 was 2.2% different from BE38 and BE41.

The phylogenetic analysis for VP6 showed that 25 out of 28 Belgian strains were closely related to each other (≤3.7%) and clustered together with other contemporary human G2P[4] strains (Fig. 6). Three other Belgian strains were found in a different cluster together with predominantly bovine or bovine-like human rotaviruses: strain RVA/Human-wt/BEL/BE67/2009/G2P[4] was most closely related to bovine-like human strain RVA/Human-wt/HUN/BP1062/2004/G8P[14], whereas BE38 and BE41 were almost genetically identical and clustered closely to bovine-like human strains RVA/Human-wt/ITA/PA169/1988/G6P[14] and RVA/Human-wt/ISR/Ro8059/1995/G6P[1], and to bovine strain RVA/Cow-tc/USA/NCDV/1971/G6P[1]. The RotaTeq vaccine strain VP6 was 2.4–5.2% different from these three Belgian bovine-like G2P[4] strains.

**Figure 7:**
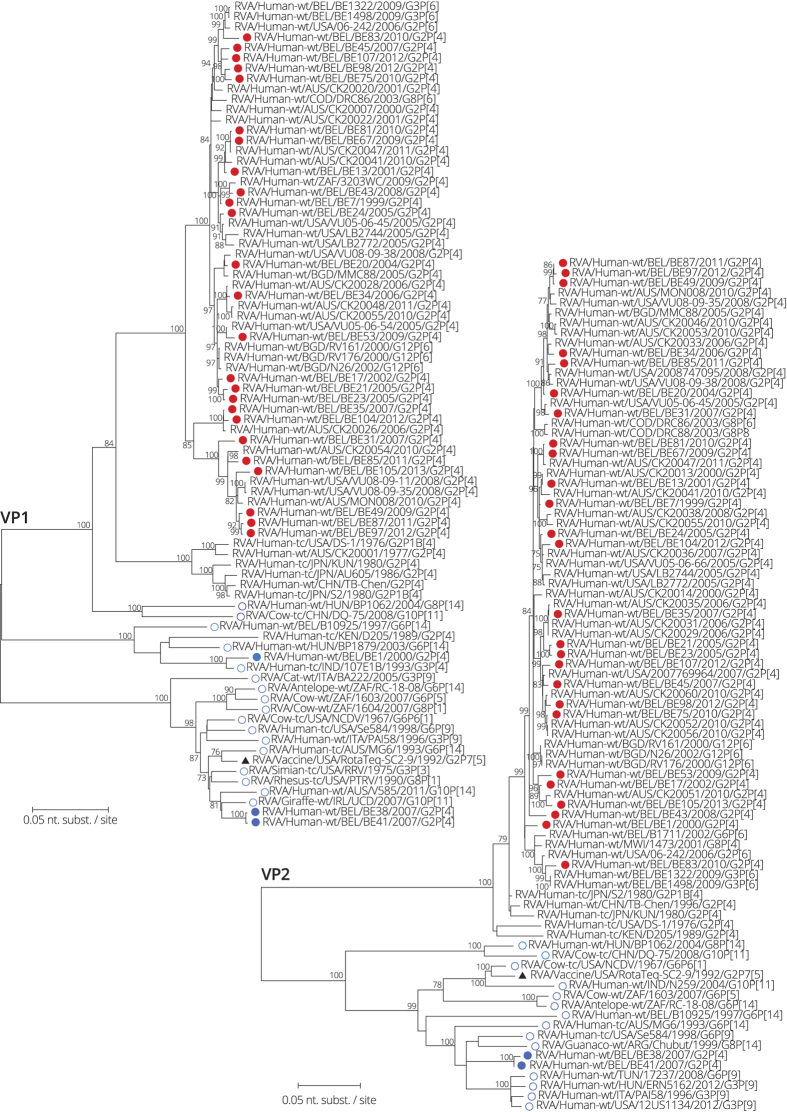
Maximum likelihood tree of VP1 and VP2. Belgian G2P[4] rotaviruses are indicated with a filled circle and colored red when clustering most closely to human DS-1-like strains and colored in different shades of blue when clustering most closely to bovine or bovine-like human rotaviruses. Bovine or bovine-like human reference strains are indicated with an open circle in different shades of blue. The RotaTeq vaccine strain is indicated by a black triangle. Only bootstrap values above 70% are shown.

The Belgian G2P[4] rotaviruses were also found in human and bovine(-like) cluster for the VP1 gene segment. Similar to the VP6 phylogenetic tree, three out of 28 Belgian strains clustered more closely to bovine and bovine-like human strains than to typical human DS-1-like rotaviruses (Fig. 7). BE38 and BE41 were almost identical and were most closely related to RVA/Giraffe-wt/IRL/UCD/2007/G10P[11] and bovine-like human strain RVA/Human-wt/AUS/V585/2011/G10P[14]. RVA/Human-wt/BEL/BE1/2000/G2P[4] clustered most closely to RVA/Human-tc/IND/107E1B/1993/G3P[4], followed at 7.2% distance by bovine-like human strain RVA/Human-wt/HUN/BP1879/2003/G6P[14]. All three Belgian DS-1-like strains belonging to animal clusters in the VP1 tree were 3.8–15.2% different to the RotaTeq vaccine VP1.

**Figure 8:**
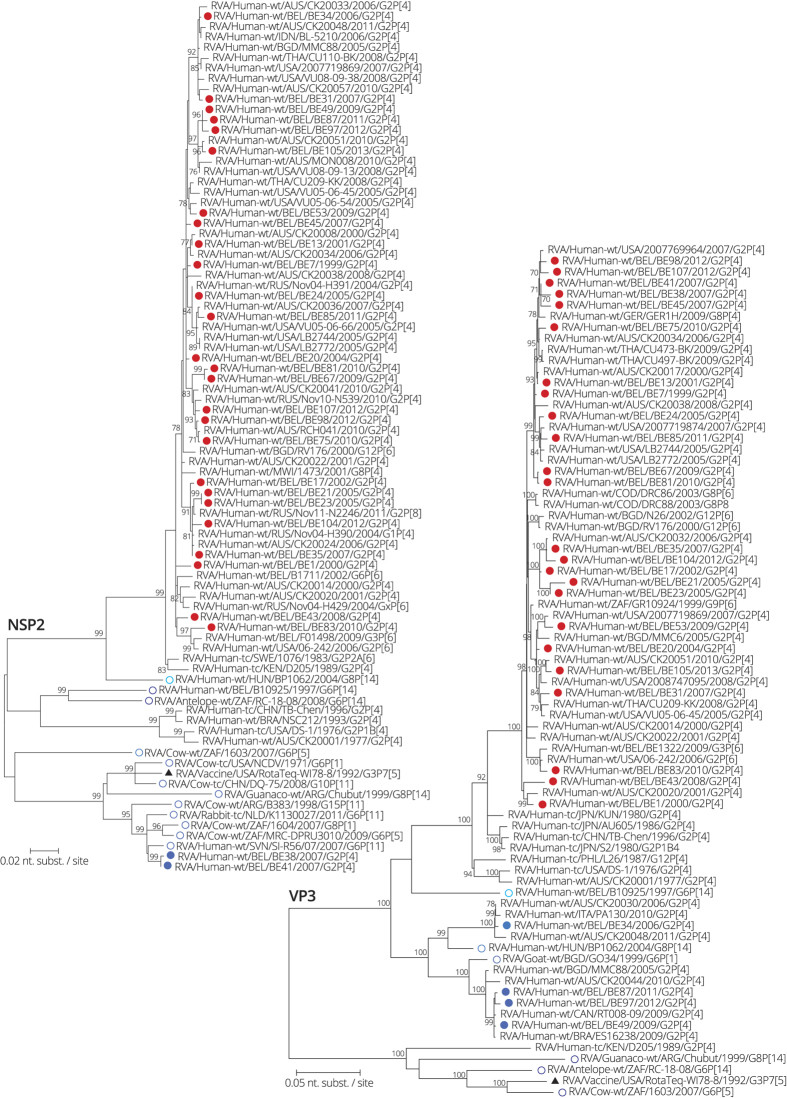
Maximum likelihood tree of NSP2 and VP3. Belgian G2P[4] rotaviruses are indicated with a filled circle and colored red when clustering most closely to human DS-1-like strains and colored in different shades of blue when clustering most closely to bovine or bovine-like human rotaviruses. Bovine or bovine-like human reference strains are indicated with an open circle in different shades of blue. The RotaTeq vaccine strain is indicated by a black triangle. Only bootstrap values above 70% are shown.

The phylogenetic trees of VP2 and NSP2 displayed a similar clustering pattern (Figs 7 and 8) with 26 out of 28 strains clustering with typical human DS-1-like strains and BE38 and BE41 clustering with bovine, bovine-like human or feline-like human strains. The RotaTeq vaccine strain was 90.3–90.0% and 93.2–93.0% similar to BE38 and BE41 for VP2 and NSP2, respectively.

For VP3, 24 out of 28 Belgian DS-1-like strains were most closely related to other typical human DS-1-like rotaviruses isolated all over the world (Fig. 8). The other four Belgian strains were found in two clusters. One cluster contained strains RVA/Human-wt/BEL/BE49/2009/G2P[4], RVA/Human-wt/BEL/BE87/2011/G2P[4] and RVA/Human-wt/BEL/BE97/2012/G2P[4], which were almost identical and were closely related to human G2P[4] strains from Brazil, Canada, Australia and Bangladesh. Caprine strain RVA/Goat-wt/BGD/GO34/1999/G6P[1] was 96.4–96.7% similar to these three Belgian strains. The other cluster contained Belgian strain RVA/Human-wt/BEL/BE34/2006/G2P[4], which was closely related to G2P[4] strains isolated in Italy and Australia. The bovine-like human strain RVA/Human-wt/HUN/BP1062/2004/G8P[14] was found at 5.2% distance from RVA/Human-wt/BEL/BE34/2006/G2P[4].

Taken together, the phylogenetic analysis for each gene segment revealed a wide variation in the genetic composition of Belgian DS-1-like rotaviruses. Twelve Belgian G2P[4] strains possessed a completely human DS-1-like genotype constellation, whereas 10 Belgian rotaviruses were most closely related to human DS-1-like strains for ten out of eleven gene segments. Four Belgian strains contained two animal derived gene segments. For BE38 and BE41 the majority of gene segments were more closely related to bovine than to human strains (Fig. 3).

## Discussion

A relatively high prevalence of G2P[4] strains has been observed in several countries where Rotarix is the mainly used rotavirus vaccine^18^,^24^,^25^. A slightly lower vaccine effectiveness has been shown against G2P[4] rotaviruses when compared to Wa-like rotaviruses (G1, G3, G4 and G9) in Belgium, which may explain the relative increase in the prevalence of G2P[4] rotaviruses in Belgium^19^,^26^. We show here that the vaccine introduction in Belgium is also temporarily associated with substantial changes in the genetic makeup of G2P[4] rotaviruses in terms of the large scale transmission of bovine-like NSP4 gene segments in the human rotavirus population (Fig. 1).

Currently, it is unclear whether the emergence of rotaviruses possessing a bovine-like NSP4 gene is a cause, a consequence or completely unrelated to the increased prevalence of G2P[4] rotaviruses after vaccine introduction. Rotaviruses containing a bovine-like NSP4 gene have been detected in other countries with rotavirus vaccination programs, such as Brazil, Australia, the USA and several countries in Sub-Saharan Africa^10^,^12^,^15^,^27^, but also in countries were no rotavirus vaccines are universally used like the Democratic republic of the Congo, Italy, Russia and Thailand^11^,^14^,^16^. It is yet unknown for how long these bovine NSP4 gene segments have been circulating in the human rotavirus population and to elucidate this, detailed large scale analyses with time-stamped animal and human DS-1-like NSP4 genes are needed. However, human rotaviruses carrying a bovine-like NSP4 gene have been detected in humans only relatively recently, suggesting that at least the two largest bovine-like NSP4 clusters observed in this study (Fig. 1) are the result of worldwide viral migration rather than of a putative selection pressure caused by the usage of the Rotarix vaccine within Belgium. Therefore, we hypothesize that it is most likely that the increased relative prevalence of G2P[4] rotaviruses as a result of universal Rotarix vaccination in Belgium also promoted the spread of G2P[4] rotaviruses with a bovine-like NSP4. Such a selective sweep has previously been reported for the VP7 gene segment of human G2P[4] strains and suggests that this is probably an important mechanism for G2P[4] rotaviruses to antigenically adapt^10^. The results of this study suggest that for NSP4 these selective sweeps may also occur in a similar way as for VP7. The fact that animal NSP4 genes are globally reported suggests that animal NSP4 genes may not pose a selective disadvantage over typically human NSP4 genes. However, adaptive immunity is less well understood for NSP4 than for VP7 and VP4 and clearly defined NSP4 antigenic epitopes are lacking. It seems likely that selective sweeps are not only restricted to NSP4 and VP7, but could potentially involve other gene segments as well.

Interspecies reassortment between bovine-like and human rotaviruses was frequently detected in Belgian G2P[4] rotaviruses, as we have found evidence for at least 8 independent interspecies reassortments in 7 different gene segments (VP6, VP1-3, NSP2, NSP4 and NSP5). For at least three clusters (two clusters in NSP4 and one in VP3) evidence was found for sustained human-to-human transmission as similar bovine-like genes segments were detected in multiple rotavirus seasons (up to five consecutive rotavirus seasons). However, our data also indicated that human-to-human transmission is rather rare for most of these human-animal reassortants, suggesting that most interspecies reassortments are probably dead-end infections. Although the three human-to-human transmission clusters are unlikely to have originated in Belgium, the interspecies reassortant dead-end infections may have originated in Belgium as they often do not cluster closely to any other known strains from the global collection (Figs 1 and 6, 7, 8). Alternatively, this could be due to undersampling or the lack of sufficient complete genome data of DS-1-like and animal rotaviruses.

Twenty-eight Belgian rotaviruses were selected for complete genome sequencing and this revealed that interspecies reassortment was not only restricted to the NSP4 gene segment, although reassortment was less often observed in gene segments other than NSP4. As the NSP4 gene encodes an enterotoxin, it be interesting to see if there were differences in clinical severity in patients infected with RVA strains with a bovine derived NSP4 gene product. Unfortunately, this information was not present. It has been shown that bovine rotaviruses are infecting humans on a regular basis. Compared to other animal rotaviruses, particularly bovine rotaviruses with a P[14] VP4 have been detected relatively frequently in humans^9^,^28^,^29^. As thus far no G2P[4] rotaviruses have been found in ruminants, humans are then most likely a mixing vessel in which interspecies reassortment occurs. It is unknown whether reassortment between human and bovine rotaviruses is restricted to certain gene segments, but it seems likely that gene segments sharing the same genotype in human and ruminant rotaviruses (VP1-VP3, VP6, NSP2 and NSP4) may reassort without great loss in fitness. For the other gene segments this is less clear and additional experiments using *in vitro* generated reassortants are needed to answer this question. However, we found two strains, BE38 and BE41, which contained six bovine gene segments, including an H3 NSP5. These two strains were genetically almost identical, isolated from the same rotavirus season and most likely epidemiologically linked to each other, indicating that a rotavirus with an H3 NSP3 is able to successfully spread from one human to another.

One of the main limitation of this study is the way samples were collected. As we did not anticipate to find such a great diversity in the NSP4 gene and to maximize genetic diversity in our selection, samples were collected based on the genetic diversity of VP7. This makes it difficult to extrapolate our findings to the Belgian G2P[4] rotavirus population as a whole. However, we do believe that our study provides a good framework for future studies to investigate the prevalence of animal gene segments in DS-1-like rotaviruses in different geographical regions and in countries with and without universal rotavirus vaccination program. Our G2P[4] samples originated from multiple hospitals across Belgium, but the impact of this on genetic diversity is probably low as Belgium is a small country. However, little is known about the impact of geographical variation on rotavirus genetic diversity at such a small scale.

Previously it has been reported that the RotaTeq vaccine strain reassorted with wild-type Wa-like strains, creating wild-type Wa-like strains possessing RotaTeq NSP2 gene segments^30^. In our dataset we did not find any evidence of reassortment with RotaTeq, despite the fact that RotaTeq is used in Belgium (approximately 15% of all administered rotavirus vaccines is RotaTeq). However, the high number of reassortants in our dataset suggest that the chance of reassortment between RotaTeq and human DS-1-like strains is not negligible, especially since the process of vaccination itself provides for ample reassortment opportunities. Therefore rotavirus surveillance involving the sequencing of complete DS-1-like genomes is especially needed in countries with a universal rotavirus vaccination program.

## Methods

Stool samples were collected at multiple hospitals in Belgium in the framework of the Belgian National Reference Center activities for rotavirus at the University Hospital Leuven, organized and approved by the WIV (Wetenschappelijk Instituut voor Volksgezondheid; Scientific Institute for Public Health) in Belgium (https://nrchm.wiv-isp.be/nl/ref_centra_labo/default.aspx). All experiments were performed according to the guidelines and regulations of the WIV. Stool samples were collected from patients hospitalized with acute rotavirus gastroenteritis, and the G-genotype and P-genotype of these samples was determined. To minimize redundant sequencing of identical NSP4 genes a preliminary phylogenetic analysis of G2 genotypes was conducted, which was used to select 89 samples that reflected the genetic diversity of the G2-genotype in every rotavirus season. Based on the phylogenetic diversity of the obtained 89 NSP4 sequences, 28 strains were selected for complete genome sequencing. In this way, our sample set is likely to reflect the overall genetic diversity of Belgian G2P[4] samples between 1999–2013, but caution should be taken with quantitative extrapolation as the samples were not randomly selected.

Stool samples were diluted once in PBS and RNA was extracted using the Qiagen Viral RNA mini kit (Qiagen) according to manufacturer’s recommendations. The NSP4 gene segment was amplified with a one-step RT-PCR kit (Qiagen) using forward primer: 5′-GGC TTT TAA AAG TTC TGT TCC-3′ and reverse primer: 5′-GGW YAC RYT AAG ACC RTT CC-3′, and the following conditions: 50 °C for 30 min, 95 °C for 15 min, followed by 35 cycles of amplification (30 sec at 94 °C, 30 sec at 45 °C, and 3 min at 72 °C), with a final extension of 10 min at 72 °C. The PCR amplicons were purified with EXO-SAP-it™ (Affymetrix) and subsequently sequenced using the same forward primer as used for the RT-PCR with the ABI PRISM™ BigDye Terminator Cycle Sequencing Reaction kit (Applied Biosystems).

The complete genome sequences for eleven strains were determined using the primers as described in Supplementary Table S1. For 17 other complete G2P[4] genomes next-generation sequencing technology was used. For this, viral RNA was first amplified using the Whole transcriptome kit (WTA2, Sigma-Aldrich) and sequence libraries were prepared using the KAPA library preparation kit (Kapa Biosystems) and sequenced in paired-end mode on a HiSeq 2500™ (Illumina) for 151 cycles. Sequences were trimmed for quality and adapters with Trimmomatic v0.32^31^ and assembled using SPAdes v3.1^32^. Rotavirus contigs were identified using Blastn^33^. Sequences have been deposited in GenBank and are available under accession numbers KR705141–KR705509. Maximum likelihood phylogenetic trees with 500 bootstrap replicates were constructed using a generalized time-reversible (GTR) model allowing for invariant sites in Mega 6^34^. For all the trees, reference strains were used, as well as a selection of contemporary strains which were genetically closely related to the Belgian G2P[4] strains under investigation.

## Additional Information

**How to cite this article**: Zeller, M. *et al.* Emergence of human G2P[4] rotaviruses containing animal derived gene segments in the post-vaccine era. *Sci. Rep.* **6**, 36841; doi: 10.1038/srep36841 (2016).

**Publisher’s note:** Springer Nature remains neutral with regard to jurisdictional claims in published maps and institutional affiliations.

## Supplementary Material

Supplementary Information
