# Erratum: Emergence of human G2P[4] rotaviruses containing animal derived gene segments in the post-vaccine era

**DOI:** 10.1038/srep39436

**Published:** 2017-01-03

**Authors:** Mark Zeller, Valerie Nuyts, Elisabeth Heylen, Sarah De Coster, Nádia Conceição-Neto, Marc Van Ranst, Jelle Matthijnssens

Scientific Reports 6: Article number: 3684110.1038/srep36841; published online: 11 14 2016; updated: 01 03 2017

The original version of this Article contained an error in the name of the first author Mark Zeller which was incorrectly given as Jelle Matthijnssens. This error has now been corrected in the PDF and HTML versions of this Article.

