# Supplementary material for: Emergence of human G2P[4] rotaviruses containing animal derived gene segments in the post-vaccine era
Source: Sci Rep. 2016 Nov 14;6:36841. doi: 10.1038/srep36841 (PMC5107926; doi:10.1038/srep36841)
Supplement: Supplementary Information [file srep36841-s1.pdf]

Emergence of human G2P[4] rotaviruses containing animal derived gene segments in the post-vaccine era

Mark Zeller, Valerie Nuyts, Elisabeth Heylen, Sarah De Coster, Nádia Conceição-Neto, Marc Van Ranst and Jelle Matthijnssens

Supplementary Table S1. Primers used to amplify all eleven gene segments of Belgian DS-1-like strains.

Gene segment	Primer name	Primer sequences
VP1	GEN_VP1Fb	5’-GGC TAT TAA AGC TRT ACA ATG GGG AAG-3'
	GEN_VP1Rb	5’-GGT CAC ATC TAA GCG YTC TAA TCT TG -3'
VP2	GEN_VP2Fc	5’-GGC TAT TAA AGG YTC AAT GGC GTA CAG-3'
	GEN_VP2_Rbc	5’-GTC ATA TCT CCA CAR TGG GGT TGG -3'
VP3	GEN_VP3Fe	5’-GGC TWT TAA AGC ART ATT AGT AGT G-3’
	GEN_VP3_2584R	5'- TGA CYA GTG TGT TAA GTT TYT AGC -3'
VP4	VP4-1-17F	5’-GGCTATAAAATGGCTTCGC-3’
	GEN_VP4_P4_2355R	5'- ACA TCC TSR ATG ACA TTC TCA C -3'
VP6	GEN_VP6F	5’-GGC TTT WAA ACG AAG TCT TC -3
	GEN_VP6R	5’-GGT CAC ATC CTC TCA CT -3’
VP7	BEG9	5’-GGCTTTAAAAGAGAGAATTTCCGTCTGG-3’
	END9	5’-GGTCACATCATACAATTCTAATCTAAG-3’
NSP1	GEN_NSP1F	5’-GGC TTT TTT TTA TGA AAA GTC TTG -3’
	GEN_NSP1R	5'-GGT CAC ATT TTA TGC TGC C -3’
NSP2	GEN_NSP2F	5’- GGC TTT TAA AGC GTC TCA G -3’
	GEN_NSP2R	5’- GGT CAC ATA AGC GCT TTC -3’
NSP3	GEN_NSP3F	5’- GGC TTT TAA TGC TTT TCA GTG -3’
	GEN_NSP3R	5’- ACA TAA CGC CCC TAT AGC -3’
NSP4	GEN_NSP4F	5’- GGC TTT TAA AAG TTC TGT TCC -3’
	GEN_NSP4R	5’- GGW YAC RYT AAG ACC RTT CC -3’
NSP5	GEN_NSP5F	5’-GGC TTT TAA AGC GCT ACA G -3’
	GEN_NSP5R	5’-GGT CAC AAA ACG GGA GT -3’
